# Diagnostic and Predictive Value of Serum Sestrin-2 and Hypoxia-Inducible Factor-1alpha in Gestational Diabetes Mellitus: A Case–Control Study

**DOI:** 10.3390/biomedicines14051036

**Published:** 2026-05-02

**Authors:** Yeliz Çeçen Dönmez, Esra Keles, İsmail Bağlar, Fatih Şanlıkan, Sahra Sultan Kara, Naile Fevziye Misirlioglu, Seyma Dumur, Oznur Dundar Akın, Aylin Yılmaz, Hafize Uzun

**Affiliations:** 1 Department of Obstetrics and Gynecology, University of Health Sciences, Kartal Dr. Lütfi Kırdar City Hospital, Istanbul 34668, Türkiye; yelizcecendonmez@gmail.com (Y.Ç.D.); ismailbg@gmail.com (İ.B.); sahracavusoglu@gmail.com (S.S.K.); 2 Department of Gynecologic Oncology, University of Health Sciences, Kartal Dr. Lütfi Kırdar City Hospital, Istanbul 34668, Türkiye; dresrakeles@gmail.com (E.K.); fatihsanlikan1@gmail.com (F.Ş.); 3 Department of Biochemistry, Faculty of Medicine, Istanbul Atlas University, Istanbul 34408, Türkiye; nailemisirlioglu@gmail.com (N.F.M.); seyma.dumur@atlas.edu.tr (S.D.); 4 Department of Obstetrics and Gynecology, Faculty of Medicine, Istanbul Atlas University, Istanbul 34408, Türkiye; oznur.akin@atlas.edu.tr; 5 Department of Perinatology, University of Health Sciences, Kartal Dr. Lütfi Kırdar City Hospital, Istanbul 34668, Türkiye; draylinyilmaz@hotmail.com

**Keywords:** gestational diabetes mellitus, Sestrin, hypoxia-inducible factor-1alpha, oxidative stress, biomarkers, insulin resistance, inflammation, pregnancy, metabolic dysregulation

## Abstract

**Background:** Gestational diabetes mellitus (GDM) is a common metabolic disorder characterized by glucose intolerance and associated with adverse maternal and fetal outcomes. Emerging evidence suggests that oxidative stress and hypoxia-related pathways may contribute to its pathophysiology. This study aimed to evaluate the diagnostic performance of serum sestrin-2 (SESN-2) and hypoxia-inducible factor-1alpha (HIF-1α) as potential biomarkers in GDM. **Methods:** In this case–control study, 100 pregnant women (50 with GDM and 50 controls) were enrolled. Serum SESN-2 and HIF-1α levels were measured using enzyme-linked immunosorbent assay (ELISA). **Results:** Patients with GDM showed significantly higher body mass index, glucose levels, glycated hemoglobin (HbA1c), insulin, and C-reactive protein (CRP) (all *p* < 0.05). SESN-2 and HIF-1α levels were significantly elevated (both *p* < 0.0001). Receiver operating characteristic (ROC) analysis showed area-under-the-curve (AUC) values of 0.799 for SESN-2 and 0.769 for HIF-1α, which increased to 0.909 when combined. Both biomarkers were independently associated with GDM in multivariable analysis. **Conclusions:** SESN-2 and HIF-1α levels are elevated in GDM and are associated with its presence. These biomarkers demonstrated moderate diagnostic performance, and their combined use improved discrimination; however, they should be considered complementary rather than standalone diagnostic tools. Given the cross-sectional design, the findings reflect associations rather than predictive relationships, and further prospective studies are required to clarify their clinical utility.

## 1. Introduction

Gestational diabetes mellitus (GDM) is a common metabolic disorder of pregnancy, defined by glucose intolerance with onset or first recognition during gestation, and is associated with adverse maternal and neonatal outcomes as well as long-term metabolic risks for both mothers and offspring [1]. Pregnancy is physiologically characterized by progressive insulin resistance, particularly in the second and third trimesters; however, in GDM, inadequate pancreatic β-cell compensation leads to hyperglycemia and metabolic dysregulation [1,2]. This condition increases the risk of complications such as preeclampsia, cesarean delivery, macrosomia, and future type 2 diabetes mellitus (T2DM), largely driven by placental hormone-mediated impairment of insulin sensitivity, underscoring the importance of early identification and risk stratification [2].

Increasing evidence indicates that oxidative stress plays a central role in GDM pathophysiology by linking hyperglycemia, inflammation, and insulin resistance. This imbalance between reactive oxygen species (ROS) production and antioxidant defenses leads to cellular damage and impaired metabolic signaling [3]. In pregnancy, oxidative stress has been associated not only with GDM but also with complications such as pre-eclampsia and placental dysfunction, with consistently elevated oxidative markers and reduced antioxidant capacity reported in affected patients [4]. Moreover, oxidative stress contributes to chronic low-grade inflammation, further impairing insulin signaling and β-cell function [1]. Sestrins (SESNs) are evolutionarily conserved proteins that maintain metabolic homeostasis by responding to stressors like DNA damage, hypoxia, and nutrient deprivation [5,6,7,8]. In vertebrates, the family includes SESN1, predominantly found in high-metabolic tissues (heart, brain, muscle), and SESN2, a sensitive sensor for dietary and environmental stress. While these proteins are critical regulators of redox balance and autophagy [9,10], their specific role as biomarkers in GDM remains under-researched, representing a significant gap in the clinical literature.

Hypoxia-inducible factor-1 (HIF-1) is a key transcription factor that orchestrates cellular responses to hypoxia by regulating genes involved in oxygen homeostasis. It is a heterodimeric protein composed of two subunits: HIF-1α and HIF-1β. In addition, hypoxia-related pathways, particularly those mediated by HIF-1α, are implicated in placental dysfunction and metabolic adaptation, highlighting the interplay between hypoxia and redox imbalance in GDM [11,12,13,14].

Importantly, similar alterations in SESN-2 and hypoxia-inducible factor-1α (HIF-1α) have been reported in obesity and preeclampsia, supporting their role as markers of oxidative stress, metabolic stress, and hypoxia-related pathways rather than disease-specific indicators [15,16,17,18,19]. These findings suggest that SESN-2 and HIF-1α may reflect broader pathophysiological processes relevant to GDM. Although their clinical significance in this context remains to be fully elucidated, these observations provide a rationale for further investigation.

In this context, the present study aimed to evaluate the clinical and diagnostic significance of serum SESN-2 and HIF-1α levels in patients with GDM and to assess their association with disease presence and underlying pathophysiological mechanisms beyond conventional glycemic indices.

## 2. Materials and Methods

### 2.1. Study Design and Ethical Approval

This case–control study was conducted at Koşuyolu High Specialization Training and Research Hospital. Pregnant women diagnosed with GDM and age-matched healthy pregnant controls were enrolled. The study protocol was approved by the Clinical Research Ethics Committee of Kartal Koşuyolu High Specialization Training and Research Hospital (Approval No: 2025/20/1265; Date: 4 November 2025). All procedures were carried out in accordance with the ethical standards of the Declaration of Helsinki, and written informed consent was obtained from all participants prior to their inclusion in the study.

### 2.2. Study Population

#### Diagnosis of Gestational Diabetes Mellitus

A post hoc power analysis was performed based on the observed difference in serum SESN-2 levels between the GDM and control groups. Using an effect size of Cohen’s d = 1.22, a two-sided alpha level of 0.05, and 50 participants per group, the achieved statistical power was 99.9%. The study included 100 pregnant women (50 with GDM and 50 controls) between 24 and 28 weeks of gestation. Participants were consecutively recruited from pregnant women attending routine antenatal care at Kartal Koşuyolu High Specialization Training and Research Hospital during the study period, following ethical approval (Date: 4 November 2025). GDM was diagnosed using a 75 g oral glucose tolerance test (OGTT) according to the International Association of Diabetes and Pregnancy Study Groups (IADPSG) criteria [20]. GDM was diagnosed if one or more of the following plasma glucose thresholds were met or exceeded:

- Fasting glucose: ≥92 mg/dL.
- 1-h glucose: ≥180mg/dL.
- 2-h glucose: ≥153mg/dL.

All participants were screened for eligibility during routine antenatal visits, and only those meeting the predefined inclusion and exclusion criteria were enrolled in the study. Participants were matched for age; however, other potential confounding variables such as body mass index (BMI) and metabolic risk factors were not matched at the design stage and were therefore adjusted for multivariable analyses. The control group consisted of pregnant women with normal glucose tolerance (NGT) who did not meet any of these criteria. Eligible participants were screened during routine clinical visits, and those meeting the inclusion criteria were invited to participate.

History of GDM was defined as a documented diagnosis of gestational diabetes in a previous pregnancy based on medical records or patient self-report.

Exclusion criteria included pregestational diabetes mellitus, chronic inflammatory diseases, active infection, autoimmune disorders, renal or hepatic dysfunction, and the use of medications that may affect glucose metabolism.

### 2.3. Data Collection and Laboratory Measurements

Blood samples for biochemical parameters and biomarker measurements (including SESN-2 and HIF-1α) were obtained after an overnight fast of at least 8 h, immediately prior to the 75 g OGTT (24–28 weeks of gestation). Blood samples were centrifuged at 3000 rpm for 10 min, and the separated serum was aliquoted and stored at −80 °C until analysis. Maternal demographic and clinical characteristics, including age, gestational age, gravidity, parity, BMI, and obstetric history, were recorded. Fasting plasma glucose, insulin, glycated hemoglobin (HbA1c), and C-reactive protein (CRP) levels were measured using standard automated laboratory methods. Insulin resistance was estimated using the homeostatic model assessment for insulin resistance (HOMA-IR), calculated as fasting insulin (µIU/mL) × fasting glucose (mg/dL)/405.

Serum SESN-2 concentrations were measured using a commercially available enzyme-linked immunosorbent assay (ELISA) kit (Human Sestrin 2, SESN2 ELISA Kit; sensitivity: 0.01 ng/mL; detection range: 0.05–15 ng/mL; Code: E3437Hu; BT Lab, Shanghai, China) according to the manufacturer’s instructions.

Serum HIF-1α concentrations were measured using a commercially available ELISA kit (Human Hypoxia-inducible Factor 1α, HIF-1α ELISA Kit; sensitivity: 0.01 ng/mL; detection range: 0.05–15 ng/mL; Code: E0422Hu; BT Lab, Shanghai, China) according to the manufacturer’s instructions.

Serum SESN-2 and HIF-1α concentrations were measured in duplicate for each sample to minimize intra-assay variability, and the mean values were used for statistical analysis. The intra-assay and inter-assay coefficients of variation were <8% and <10%, respectively, in accordance with the manufacturer’s specifications.

### 2.4. Statistical Analysis

Statistical analyses were performed using GraphPad Prism version 8.0.1. Normality was assessed using the Shapiro–Wilk test. Continuous variables are presented as the mean ± SD or median [min–max], and categorical variables as *n* (%). Between-group comparisons were performed using the independent-sample *t*-test or Mann–Whitney U test, and categorical variables using the chi-square or Fisher’s exact test. Receiver operating characteristic (ROC) analysis was used to evaluate diagnostic performance. Area under the curve (AUC) with 95% confidence intervals (CIs), optimal cut-off values (Youden index), sensitivity, specificity, positive predictive values (PPVs), and negative predictive values (NPVs) were calculated. ROC curves were compared using the DeLong test. Univariable and multivariable logistic regression analyses were performed to identify variables independently associated with GDM. Variables were selected based on clinical relevance and/or significant univariable associations. Results are presented as odds ratios (ORs) with 95% CIs. Multicollinearity was assessed using variance inflation factor (VIF), and model calibration using the Hosmer–Lemeshow test. A post hoc power analysis indicated adequate power for the primary outcome. A *p* value < 0.05 was considered statistically significant.

## 3. Results

**Figure 1:**
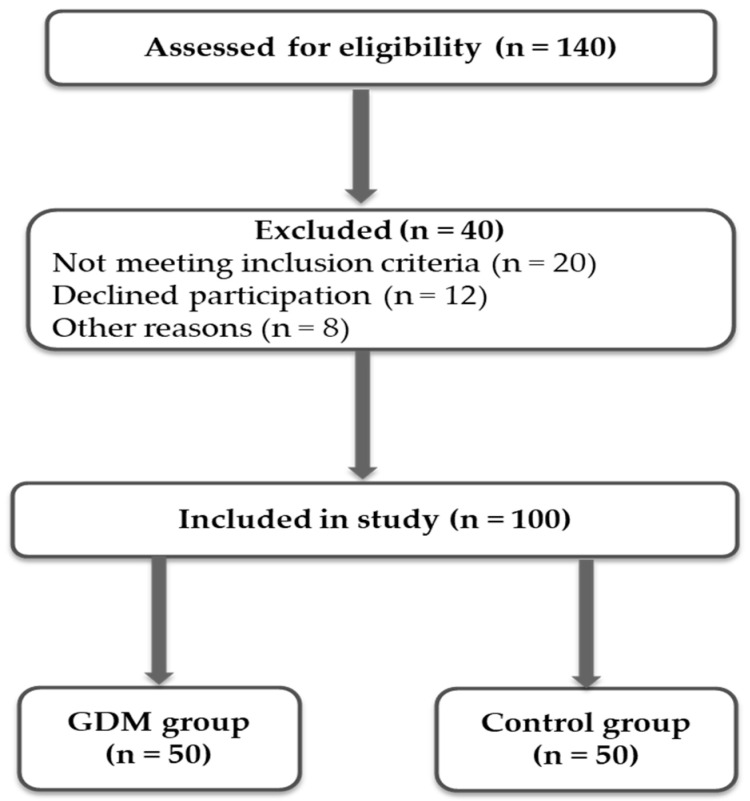
Flow diagram of participant selection and group allocation.

A flow diagram illustrating participant selection and exclusion is presented in Figure 1. Baseline maternal demographic and clinical characteristics were largely comparable between the control and GDM groups (Table 1). No statistically significant differences were observed in age, gestational age at sampling, gravidity, parity, abortion history, or height (all *p* > 0.05). In contrast, body weight and BMI were significantly higher in the GDM group (*p* = 0.004 and *p* < 0.001, respectively). In addition, previous GDM was more frequent, whereas smoking rates were lower in GDM patients (both *p* < 0.05), indicating a distinct baseline metabolic risk profile.

A clear deterioration in metabolic and inflammatory parameters was evident in the GDM group (Table 2). Fasting glucose, OGTT values at 1 and 2 h, HbA1c, insulin, and CRP levels were all significantly elevated (all *p* < 0.05), consistently with dysglycemia and systemic inflammation. Conversely, liver enzymes, protein parameters, and hematological indices remained largely comparable between groups. Creatinine levels were modest but significantly higher in the GDM group (*p* = 0.025).

Serum SESN-2 and HIF-1α concentrations were markedly elevated in patients with GDM compared to controls (Table 3), with both biomarkers reaching high statistical significance (*p* < 0.001). These findings suggest activation of oxidative stress and hypoxia-related pathways in GDM.

Evaluation of diagnostic performance demonstrated that both SESN-2 and HIF-1α had good discriminatory capacity for identifying GDM (Table 4). The AUC for SESN-2 was 0.799 (95% CI: 0.705–0.878), and for HIF-1α it was 0.769 (95% CI: 0.670–0.862), with both *p* < 0.001. At the optimal cut-off value (6.848 ng/mL), SESN-2 showed a sensitivity of 78.0% and specificity of 74.0%. Similarly, HIF-1α demonstrated a sensitivity of 72.0% and specificity of 74.0% at a cut-off value of 7.222 ng/mL. The combined biomarker model yielded a higher AUC of 0.909 (95% CI: 0.841–0.960), indicating improved discrimination. The combined model achieved a sensitivity of 78.0% and specificity of 92.0%, reflecting enhanced diagnostic performance. Pairwise comparisons using the DeLong test showed that the combined model performed significantly better than the individual biomarkers (*p* < 0.001).

Logistic regression analyses identified multiple predictors of GDM (Table 5). In univariable analysis, BMI, HbA1c, CRP, SESN-2, and HIF-1α were significantly associated with GDM. After multivariable adjustment, BMI and CRP remained significant clinical predictors, whereas SESN-2 and HIF-1α remained independently associated with GDM (all *p* < 0.05), supporting their potential additive value in risk assessment.

Correlation analyses performed within the GDM group revealed no significant associations between SESN-2 or HIF-1α levels and conventional metabolic or clinical parameters, including glycemic indices, insulin, HOMA-IR, BMI, CRP, and neonatal birth weight (all *p* > 0.05). This suggests that these biomarkers may reflect distinct biological mechanisms not captured by routine measures.

Perinatal outcomes were comparable between groups, with no statistically significant differences in gestational age at delivery, mode of delivery, neonatal birth weight, or Apgar scores at 1 and 5 min (all *p* > 0.05). These findings indicate that GDM was not associated with adverse short-term obstetric or neonatal outcomes in this cohort.

## 4. Discussion

The present study shows that serum SESN-2 and HIF-1α levels are significantly elevated in patients with GDM and are associated with GDM beyond conventional metabolic parameters. Notably, both biomarkers remained independently associated with GDM in multivariable analysis, even after adjustment for established risk factors such as BMI and CRP. These findings suggest that SESN-2 and HIF-1α may reflect distinct pathophysiological pathways not fully captured by traditional glycemic indices or inflammatory markers.

Importantly, similar alterations in SESN-2 and HIF-1α have been reported in conditions such as obesity and preeclampsia, supporting their role as markers of oxidative stress and hypoxia-related pathways rather than disease-specific indicators [15,16,17,18,19]. These observations suggest that SESN-2 and HIF-1α reflect shared pathophysiological processes across metabolic and pregnancy-related conditions, and their clinical relevance in GDM may lie in providing complementary, rather than disease-specific, information.

The findings of the present study are consistent with accumulating evidence indicating that GDM is characterized by a state of heightened oxidative stress and placental dysfunction. Previous experimental and clinical studies have demonstrated that GDM induces a chronic hypoxic intrauterine environment accompanied by increased inflammatory responses and altered placental vascular development [21]. Elevated expression of HIF-1α has been observed in placental tissues under diabetic conditions, suggesting that hypoxia signaling is a key adaptive and pathological response in GDM.

Given that biomarker measurements and GDM diagnosis were performed concurrently, the present findings reflect associations rather than temporal or predictive relationships. The observed elevations in SESN-2 and HIF-1α likely represent responses to hyperglycemia-induced oxidative stress and hypoxia rather than preceding events.

From a mechanistic standpoint, oxidative stress and hypoxia appear to act synergistically in disrupting placental homeostasis. Excessive production of ROS not only damages cellular structures but also contributes to mitochondrial dysfunction and impaired energy metabolism, thereby exacerbating metabolic dysregulation in GDM [22]. Moreover, oxidative stress has been shown to influence key metabolic signaling pathways, including AMP-activated protein kinase (AMPK) and mechanistic target of rapamycin (mTOR), linking redox imbalance directly to altered nutrient sensing and placental function [23]. These mechanisms provide a plausible biological explanation for the elevated SESN-2 levels observed in the present study, given the established role of SESN proteins in cellular stress responses and redox regulation.

Hypoxia-related signaling mediated by HIF-1α has also been implicated in metabolic reprogramming within the placenta. Under hypoxic conditions, HIF-1α promotes a shift from oxidative phosphorylation to anaerobic glycolysis, thereby contributing to metabolic inefficiency and altered glucose handling [24]. This metabolic adaptation may further aggravate insulin resistance and glucose intolerance, reinforcing the central role of hypoxia in GDM pathophysiology. In addition, hypoxia-driven pathways have been linked to trophoblast dysfunction and apoptosis, which may compromise placental development and function [25].

The interaction between oxidative stress and inflammation represents another key component of GDM. Oxidative stress can trigger inflammatory cascades that impair placental permeability and promote a hypoxic microenvironment, further amplifying metabolic and vascular disturbances [26]. This bidirectional relationship may explain why traditional inflammatory markers alone are insufficient to fully capture the complexity of GDM, highlighting the added value of biomarkers such as SESN-2 and HIF-1α.

Taken together, these findings support a model in which GDM is driven by an interconnected network of oxidative stress, hypoxia signaling, mitochondrial dysfunction, and inflammation. Within this framework, the simultaneous elevation of SESN-2 and HIF-1α likely reflects activation of complementary stress-response pathways. During pregnancy, metabolic demands are substantially elevated, and the precise regulation of glucose homeostasis becomes fundamental for maintaining maternal–fetal metabolic balance [27]. Notably, the absence of strong correlations between these biomarkers and conventional metabolic parameters further suggests that they represent independent dimensions of disease biology, rather than merely secondary markers of hyperglycemia.

From a clinical perspective, SESN-2 and HIF-1α are not intended to replace established diagnostic methods such as OGTT; rather, they may provide complementary information by reflecting underlying biological processes not captured by routine markers. Their lack of strong correlation with conventional parameters supports their role as indicators of distinct pathophysiological pathways. While OGTT remains the gold standard for GDM diagnosis, it primarily captures glycemic status at a single time point and does not provide insight into the biological processes driving disease development. In this context, SESN-2 and HIF-1α may contribute to a more comprehensive characterization of GDM; however, their clinical applicability remains to be established.

### 4.1. Strengths

Despite these limitations, this study has several notable strengths. To the best of our knowledge, it is among the first clinical studies to evaluate serum SESN-2 levels in patients with GDM, providing novel insight into oxidative stress-related mechanisms in pregnancy. The simultaneous assessment of SESN-2 and HIF-1α allows a more comprehensive evaluation of the interplay between oxidative stress and hypoxia signaling pathways. The use of a well-defined case–control design with standardized diagnostic criteria and detailed biochemical profiling strengthens the methodological rigor of the study. In addition, the application of advanced statistical approaches, including ROC analysis and multivariable logistic regression, enabled robust assessment of diagnostic performance and independent associations. Finally, the observation that these biomarkers remain significant beyond conventional metabolic parameters supports their potential role as complementary indicators of underlying pathophysiological processes.

### 4.2. Limitations

Several limitations should be acknowledged. The relatively small sample size and single-center design may limit generalizability. In addition, the cross-sectional nature of the study precludes causal inference. Although serum biomarkers were assessed, underlying mechanistic pathways and placental tissue expression were not directly evaluated. Potential confounders such as dietary intake, physical activity, and glycemic variability were not assessed, and matching was limited to age; although adjustments were made in multivariable analyses, residual confounding cannot be excluded. No a priori sample size calculation was performed; although post hoc power analysis indicated adequate power for the primary outcome, the sample size may still limit the robustness of multivariable and ROC analyses. Furthermore, no internal or external validation of the ROC models was conducted, and no correction for multiple comparisons was applied, which may have increased the risk of type I error. Given these limitations and the lack of prior clinical studies on SESN-2 in GDM, the findings should be interpreted as hypothesis-generating.

### 4.3. Future Directions

Future studies should aim to validate these findings in larger, multicenter cohorts with diverse populations to enhance generalizability. Longitudinal studies are particularly needed to determine whether SESN-2 and HIF-1α have true predictive value for the development of GDM, and to assess their temporal dynamics throughout pregnancy.

## 5. Conclusions

In conclusion, the findings of this study indicate that serum SESN-2 and HIF-1α levels are elevated in patients with GDM and are independently associated with the presence of the disease beyond conventional metabolic markers. Although both biomarkers demonstrated moderate discriminatory performance, their combined use improved overall discrimination. These results suggest that SESN-2 and HIF-1α may serve as complementary indicators reflecting underlying oxidative stress and hypoxia-related pathways rather than standalone diagnostic tools. Given the cross-sectional design, these findings should be interpreted as associations rather than causal or predictive relationships. Further large-scale, prospective studies are required to validate these observations.

## Figures and Tables

**Table 1:** Baseline maternal demographic and clinical characteristics of the study groups.

Variable	Control Group (*n* = 50)	GDM Group (*n* = 50)	*p* Value
Age (years)	30.48 ± 6.07	32.14 ± 5.30	0.148
Gestational age at sampling (weeks)	26.00 [24.00–38.00]	27.50 [16.00–34.00]	0.106
Gravida *n* (range)	2 [1–7]	3 [1–9]	0.089
Parity *n* (range)	1 [0–3]	1.5 [0–5]	0.068
Height (cm)	162 [152–176]	160 [156–177]	0.343
Weight (kg)	71.05 ± 9.66	78.73 ± 14.59	0.004
Body mass index (kg/m^2^)	26.93 ± 3.28	30.87 ± 5.20	<0.001
Smoking, *n* (%)	8 (16)	2 (4)	0.046
Previous GDM, *n* (%)	0 (0)	6 (12)	0.012

Continuous values are presented as mean ± standard deviation or median [minimum–maximum].

**Table 2:** Biochemical, metabolic, and hematological parameters of the study groups.

Variable	Control Group (*n* = 50)	GDM Group (*n* = 50)	*p* Value
Fasting glucose (mg/dL)	84.00 [70.00–95.00]	97.00 [73.00–218.0]	<0.001
OGTT. 1-h (mg/dL)	133.6 ± 23.29	168.0 ± 35.85	<0.001
OGTT. 2-h (mg/dL)	103.0 ± 20.63	138.7 ± 33.52	<0.001
HbA1c (%)	5.3 [4.6–6.0]	5.7 [4.6–7.6]	0.0002
Insulin (µIU/mL)	6.0 [4.6–79.0]	12.6 [5.5–128.4]	0.040
HOMA-IR	0.0 [0.0–16.4]	0.0 [0.0–31.1]	0.871
CRP (mg/L)	1.5 [0.0–14.6]	11.5 [0.0–49.8]	0.001
Total protein (g/dL)	6.3 [5.4–7.7]	6.3 [5.1–7.7]	0.725
Albumin (g/dL)	3.6 [2.6–4.7]	3.6 [2.5–4.7]	0.674
AST (U/L)	18.0 [12.0–75.0]	18.0 [10.0–40.0]	0.566
ALT (U/L)	13.0 [5.0–92.0]	14.0 [7.0–43.0]	0.942
Urea (mg/dL)	14.7 [7.3–30.8]	15.7 [2.3–32.0]	0.599
Creatinine (mg/dL)	0.49 [0.33–0.77]	0.52 [0.35–0.87]	0.025
Uric acid (mg/dL)	3.7 [2.3–7.2]	3.6 [1.7–10.0]	0.756
Hemoglobin (g/dL)	11.3 ± 1.2	11.6 ± 1.3	0.197
White blood cell count (×10^9^/L)	10.3 ± 2.5	9.9 ± 2.8	0.397
Platelet count (×10^9^/L)	259.9 ± 60.4	242.3 ± 76.4	0.162
Neutrophil count (×10^9^/L)	7.5 [3.0–13.4]	6.9 [3.6–6.02]	0.184
Lymphocyte count (×10^9^/L)	1.9 [1.0–4.1]	1.9 [0.7–3.30]	0.986

Continuous values are presented as mean ± standard deviation or median [minimum–maximum]. OGTT, oral glucose tolerance test; HbA1c, glycated hemoglobin; HOMA-IR, Homeostatic Model Assessment for Insulin Resistance; CRP, C-reactive protein; AST, aspartate aminotransferase; ALT, alanine aminotransferase.

**Table 3:** Serum SESN-2 and HIF-1α levels in the study groups.

Biomarker	Control Group (*n* = 50)	GDM Group (*n* = 50)	*p* Value
Sestrin-2 (ng/mL)	5.50 ± 2.01	8.41 ± 2.72	<0.000
HIF-1α (ng/mL)	6.10 ± 2.96	8.94 ± 2.84	<0.000

Continuous values are presented as mean ± standard deviation. HIF-1α, hypoxia-inducible factor-1alpha.

**Table 4:** Diagnostic performance of serum sestrin-2 and HIF-1α for identifying gestational diabetes mellitus.

Biomarker	AUC (95% CI)	Cut-Off	Sensitivity (%)	Specificity (%)	PPV (%)	NPV (%)	*p* Value
Sestrin-2 (ng/mL)	0.799 (0.705–0.878)	6.848	78.0	74.0	75.0	77.1	<0.001
HIF-1α (ng/mL)	0.769 (0.670–0.862)	7.222	72.0	74.0	73.5	72.5	<0.001
Combined model ^†^	0.909 (0.841–0.960)	0.654	78.0	92.0	90.7	80.7	<0.001

† Combined model: derived from multivariable logistic regression including SESN-2 and HIF-1α. Cut-off value represents the predicted probability determined by the Youden index. AUC: area under the curve; CI: confidence interval; PPV: positive predictive value; NPV: negative predictive value; HIF-1α, hypoxia-inducible factor-1alpha.

**Table 5:** Logistic regression analysis for predictors of gestational diabetes mellitus.

Variable	Univariable OR	95% CI	*p* Value	Multivariable OR	*p* Value
Age (Year)	1.053	0.982–1.130	0.148	1.197	0.608
BMI (kg/m^2^)	1.251	1.107–1.414	**<0.001**	2.889	**0.027**
HbA1c (%)	26.309	3.431–201	**0.002**	2.324	0.104
Insulin (µIU/mL)	1.013	0.980–1.047	0.451	1.237	0.566
CRP (mg/L)	1.448	1.146–1.829	**0.002**	19.041	**0.010**
SESN-2 (ng/mL)	1.662	1.337–2.067	**<0.001**	5.658	**<0.001**
HIF-1α (ng/mL)	1.396	1.188–1.641	**<0.001**	4.298	**<0.001**

Bold values indicate statistical significance (*p* < 0.05). Multivariable OR reported per 1 standard deviation unit: missing values imputed with median. Multivariable model includes all 7 variables simultaneously (*n* = 100). OR: odds ratio; CI: confidence interval; BMI, body mass index; HbA1c, glycated hemoglobin; CRP, C-reactive protein; SESN, sestrin; HIF-1α, hypoxia-inducible factor-1alpha.

## Data Availability

The datasets generated and/or analyzed in the current study are available from the corresponding author upon request. All data supporting the findings of this study are included within the article.
